# Association between the location of social medical insurance and social integration among China’s elderly rural migrants: a nationwide cross-sectional study

**DOI:** 10.1186/s12889-023-16956-2

**Published:** 2023-10-27

**Authors:** Xiaojie Ma, Wenjia Feng, Chaojun Shi, Yifan Wang, Qianqian Gao, Weiqin Cai, Hongqing An, Qi Jing, Runguo Gao, Anning Ma

**Affiliations:** 1 School of Public Health, Weifang Medical University, Weifang, 261000 China; 2 School of Management, Weifang Medical University, Weifang, 261000 China; 3 Institute of Public Health Crisis Management, Weifang Medical University, Weifang, China

**Keywords:** Elderly rural migrants, Social medical insurance, Social integration

## Abstract

**Background:**

Universal social medical insurance coverage is viewed as a major factor in promoting social integration, but insufficient evidence exists on the integration of elderly rural migrants (ERM), generally aged 60 years and above, in low- and middle-income countries. To address this problem, we explore the relationship between the location of social medical insurance (SMI), such as a host city, and social integration in the context of Chinese ERM.

**Methods:**

This study is based on data from the 2017 National Internal Migrant Dynamic Monitoring Survey in China. The study participants were Chinese ERM. An integration index was constructed to measure the degree of social integration in a multi-dimensional manner using a factor analysis method. This study used descriptive statistics and one-way analysis of variance to explore the differences in social integration between ERM with SMI from host cities and hometowns. Stepwise multiple linear regression analysis was used to test the correlation between SMI location and social integration level in the overall sample. Finally, the results were verified by propensity score matching.

**Results:**

It was found that 606 (18.2%) of the insured ERM chose host city SMI, while 2727 (81.8%) chose hometown SMI. The level of social integration was lower among ERM with hometown SMI (-1.438 ± 32.795, *F* = 28.311, p ≤ 0.01) than those with host city SMI (6.649 ± 34.383). Among the dimensions of social integration, social participation contributed more than other factors, with a contribution rate of 45.42%. Host city SMI increased the probability of the social integration index by 647% among ERM (k-nearest neighbor caliper matched (*n* = 4, caliper = 0.02), with a full sample ATT value of 6.47 (T = 5.32, SE = 1.48, *p* < 0.05)).

**Conclusions:**

ERM with host city SMI have a higher social integration level than those with hometowns SMI. That is, host city SMI positively affects social integration. Policymakers should focus on the access of host city SMI for ERM. Removing the threshold of host city SMI coverage for ERM can promote social integration.

## Background

In China, the world’s largest developing country, the number of migrants reached 380 million in 2020 and continues to climb. The size and nature of China’s migrant population differs significantly from that of developed countries [1]. The form of migration is predominantly domestic. People with rural households account for 82.09% of the total migrants. Among them, the number of elderly migrants is growing, increasing from 5.03 million in 2000 to 18 million in 2020. The main reasons for migration are to provide for future generations (43%), old age (25%), and employment (23%) [2]. The ERM is the hardest hit in the urbanization process and their well-being is of concern. They face numerous social integration problems, including inadequate access to economic resources, healthcare, and social services; lack of social support networks; discrimination in public services; stress related to family conflict and caregiving; social isolation; and unmet mental health needs [3, 4].

The ERM in China have a high degree of residential stability and a strong intrinsic need to integrate into their host cities; however, their ability to integrate is weak. Social integration is a term used to describe the ability of migrants to participate socially, culturally, economically and politically in the host city. It is a multidimensional concept [5]. Their social integration into host cities affect their physical and mental health [6–9]. Being both migrants and elderly, their physical state and social skills are in decline. While living in an unfamiliar city, they often face problems such as social capital reconstruction, reduced social support, and difficulties in social integration, making them the social group that finds it the most difficult to integrate in host cities [10, 11]. Given the current situation of social integration of ERM, they face various obstacles in terms of economic integration, cultural and social adaptation, structural integration, identity, and other factors [12, 13]. Compared to other migrant groups, the poor social integration of ERM is of greater concern.

The issue of SMI coverage for the migrant population has long been a concern for policymakers. There are regional differences in insurance policies in the various influx areas, but in general they support conditional coverage of the migrant population. Migrants could enroll in host city SMI [14, 15]. For example, those who have a permanent job can join urban employee-based basic medical insurance purchased by their employer. Other migrants can apply for a residence permit and join the new rural cooperative medical scheme and the urban resident based basic medical insurance in host cities. The following conditions must be met in order to apply for a residence permit. Migrants must leave their hometown to live in the host city for more than 6 months and must meet one of the conditions of legal and stable employment, legal and stable residence and continuous education. Once the host city SMI has been secured, migrants will enjoy the same medical reimbursement policy as local residents. Reimbursement reduces the financial pressure on migrants. The host city SMI also promotes the use of medical services [16]. Migrants can be insured in their hometown if they cannot be insured in host cities. Some choose to return to their hometown to access health services and receive reimbursement. Others choose to stay in the host city and claim reimbursement for medical services. Chinese policymakers have encouraged the development of cross-territory medical reimbursement. In 2018, the National Health Insurance Administration improved the off-site filing system to enable the direct settlement of inpatient expenses in different locations. For example, migrants who wish to use hospital services in host city must register in advance with the Health Protection Bureau. The off-site filing system refers to the registration of migrants characteristics and enrolment information with the National Health Insurance Administration through online or offline means. Cross-location direct settlement means to enjoy the reimbursement policy directly in the host city without returning to the hometown. In this way, the migrant population can enjoy the subsidy of SMI when they are discharged from hospitals for check-up. In 2020, the “Notice of the Ministry of Finance of the National Health Insurance Administration on Promoting the Pilot of Cross-Province Direct Settlement of Outpatient Expenses” was issued to promote the direct settlement of outpatient expenses in different places [17]. In other words, the outpatient expenses in host city are subsidized by the insurance. In 2021, the “Notice of the National Health Insurance Administration and the Ministry of Finance on Accelerating the Work of Cross-Provincial Direct Settlement of Outpatient Expenses” will be issued to orderly carry out the pilot cross-provincial direct settlement of expenses for five types of outpatient chronic diseases, as well as to accelerate the construction of the national health insurance information platform [18]. In other words, the direct settlement of outpatient expenses for certain chronic diseases has entered a pilot program. In 2022, the “Rules for Handling Cross-Provincial Direct Settlement of Basic Medical Insurance Expenses” was issued to further promote cross-provincial direct settlement [19]. This means that migrants are directly subsidized for SMI when they pay for health care in all areas of the province where they are registered.

For migrants, there are differences in insurance policies across China. In some areas, there are barriers to SMI policies. This may affect the incentives for migrants to enroll in coverage in the host city. Conditional coverage policies may reduce the accessibility of health resources for some migrants. This in turn affects their well-being. And the empirical analysis on the correlation between location of SMI and social integration is insufficient in previous studies, especially for the vulnerable population of ERM in developing countries. The purpose of this study is to explore the relationship between location of SMI and social integration. This paper attempts to test this hypothesis by comparing the difference in the level of social integration between the SMI of the host city and the SMI of the hometown using nationwide data to provide more research evidence in this area.

Previous studies have shown that the primary factors influencing social integration include policies, health systems, labor contracts, homeland culture and customs, education level, social status, age, and gender [11, 20]. For example, discriminatory policies in host city can inhibit social integration [21, 22]. It is generally accepted that the greater a person’s age, the more difficult social integration is [23]. Studies have shown that extending SMI coverage is recommended by the World Health Organization as a typical means of promoting social integration [24]. This study takes an ERM as the study population and analyzes whether the SMI of host city promotes social integration.

If ERM with host city SMI have a higher level of social integration than those with hometown SMI, policymakers could focus on the access of host city SMI for ERM. Removing the threshold for host city SMI facilitates the ERM’ welfare benefits as natives. On this basis, the cumbersome process of returning to their hometown for reimbursement can be avoided. This is essential to promote this ERM’s social integration. The aim is to investigate whether a difference exists in the social integration level between SMI of host cities and that of hometowns among the ERM, as well as the impact of SMI of host cities on the social integration for ERM.

## Methods

### Data source

The data for this study were derived from Volume A of the 2017 National Internal Migrant Dynamic Monitoring Survey, which was funded and organized by the National Population and Family Planning Commission of the People’s Republic of China [25]. This database was chosen because it is the most recent national level data survey on migrants and contains basic social-economic characteristics, social health insurance, and especially social integration of migrants. The survey used a stratified, multi-stage, size-proportional PPS sampling method. The scope covers 31 provinces (municipalities and districts) and Xinjiang Production and Construction Corps areas across China, with a certain degree of reliability and validity [26]. The survey defines the migrant population as “the male and female migrant population aged 15 years and over (born in April 2002 and before) who have resided in host city for 1 month or more and who do not have a household registration in the district (city or county)” [27].

### Participants

Participants in the survey are male and female migrants aged 60 and above (born in 1957 or before) with rural household registration who have resided in host city for 1 month or more, and who are not household residents of the host city, and who are covered by social health insurance. The sample size for this inclusion criterion was 3333 participants. ERM were selected because they are an important part of the internal migrants. The integration of ERM in the host cities was a concern.

### Variables

#### Variable selection and measurement

The dependent variable in this study is social integration, and the independent variable is the location of SMI. Individual, family-type, and social-type characteristics that affect social integration were used as control variables in conjunction with previous studies (see Table 1 for details).

**Table 1:** Variable selection (*n* = 3333)

**Variable class**	**Variable name**	**Variable declaration**	**Total sample**		**SMI of host city**		**SMI of hometown**	
			**Mean**	**Standard deviation**	**Mean**	**Standard deviation**	**Mean**	**Standard deviation**
**Dependent variable**	Social integration	10 social integration indicators were selected for factor analysis and the comprehensive score was obtained	-3.3 × 10^–5^	33.220	6.469	34.383	-1.441	32.799
**Independent variable**	Location of SMI	hometown = 0, host city = 1	0.18	0.386	-	-	-	-
**Social variables**	Gender	male = 1, female = 2	1.40	0.490	1.42	0.495	1.39	0.489
	Education level	Primary school and below = 1,Junior middle school = 2, high school and above = 3	1.42	0.628	1.36	0.591	1.43	0.635
	Health status	Health = 1, unhealthy = 2	1.22	0.416	1.31	0.461	1.21	0.404
**Household variables**	Marital status	Marriage remarried cohabitation = 1, unmarried, divorced or widowed = 2	1.16	0.368	1.17	0.379	1.16	0.365
	Average household monthly income	3000 below for = 1,3000–5999 for = 2, 6000–8999 for = 3, and above 9000 = 4	1.87	1.043	1.66	0.942	1.91	1.059
	Short-stay intention	Unwilling = 1, willing = 2	1.20	0.402	1.11	0.318	1.22	0.416
	Settlement intention	Unwilling = 1, willing = 2	1.63	0.483	1.51	0.500	1.66	0.475
**Social variables**	Term of a labour contract	No fixed term = 1, with fixed term = 2	1.78	0.196	1.76	0.200	1.78	0.195
	Reasons for unemployment	Family factor = 1, occupational environment factor = 2, personal physical, psychological and other factors = 3, non-labor force stage and other = 4	2.74	0.829	2.82	0.782	2.72	0.839
	Employment identity	Employee = 1, employer or self-operator = 2	1.43	0.325	1.43	0.289	1.44	0.333
	health record	Yes = 1, no = 2, unclear = 3	1.85	0.639	1.74	0.725	1.87	0.616
**Migrant characteristic**	Migrant range	trans-provincial = 1, Inter-city within the province = 2, The city crosses the county = 3, cross border = 4	1.80	0.782	1.71	0.754	1.83	0.787
	Migrant time	Below 5 years = 1,5–10 years = 2, above 10 years = 3	1.92	0.868	2.26	0.855	1.84	0.852
	Migration reason	employment = 1, family members move = 2, relocation and move = 3,to grow old = 4, other = 5	1.70	0.928	1.79	1.042	1.68	0.900

#### Dependent variable

Based on the indicator system proposed by Hader from Immigration Policy Lab, variables were selected to measure social integration [28]. The variables were synthesized in terms of social participation, governance participation, psychological identity, and customs participation, and a total of 17 indicators were selected. These indicators are: participation in labor unions, volunteer associations, classmates’ associations, hometown associations, hometown chambers of commerce; management advice for their community and company; policy suggestions responded to the government; online participation in state and social governance; volunteer activities; activities of the party organization; fondness for host city; concern about the host city; perceived attitudes of the host city; the importance of hometown customs; and the differences in customs and habits with the host city; whether to apply for temporary residence permit; whether to apply for a social security card. Through factor analysis, four male factors were extracted-social participation, governance participation, psychological identity, and customs participation. There are four dimensions constructed by factor analysis as shown in Table 2. Based on this, the four common factors (F1-F4) were rotated using the maximum variance method. The proportion of variance contribution of each common factor was used as the weight to calculate the overall social integration score.

**Table 2:** Social integration index

**Common factor (variance contribution ratio)**	**Variable**
**F1 Social participation (45.42%)**	Participate in the hometown associations
	Temporary residence permit
**F2 Governance participation (19.61%)**	Management advice for their community and company
**F3 Psychological identity (20.61%)**	I like where I live right now
	I focus on the change in my host city
	I’m very willing to fit in with the local people
	I feel local people are willing to accept me as one of them
	I feel like I’m already a native
**F4 Customs participation (20.61%)**	It is more important to follow the customs
	My hygiene habits are quite different from those of the local citizens

The result was consistent with Yang’s and Xia’s studies on migrants’ social integration in China [27, 29] (see Table 2 for details). F1 is measured by two variables representing social activities and institutional participation, named “social participation” [30, 31]; F2 is measured by indicators representing participation in local community, village affairs and workplace management and other types of social government participation, named “governance participation” [4]; F3 is measured by the five indicators of the representative’s enjoyment of the host city and psychological identification, named “psychological identity” [32]; and F4 is measured by the two indicators of the representative’s identification with and assimilation of the culture, customs, and habits of the host city, named “customs participation [33, 34]”.

#### Independent variables

The independent variable here is the location of SMI, that is, the place where SMI availed. SMI of the host city is scored “1”; SMI of the hometown is “0”. Participation in any of the following is considered as participation of SMI: “the new rural cooperative medical scheme”, “urban and rural residents’ cooperative medical insurance”, “urban resident based basic medical insurance”, “urban employee-based basic medical insurance”, and “free medical treatment”.

#### Control variables

Based on relevant studies, the control variables here are divided into individual characteristic, household, social, and migrant characteristic variables. The individual variables include gender, education level, and health status. Household variables include marital status, average household monthly income, short-stay intention and settlement intention. Social variables include term of labour contract, reason for unemployment, employment identity, and establishment of a health record [35]. The migration variables include migration range, reasons for migration, and region of migration.

#### Methods

The study employed IBM SPSS (25.0) and STATA (17.0) to clean, describe, analyze, and process the data; we then used Factor Analysis to construct a variable system for the ERM’s social integration index. One-way analysis of variance was conducted to check whether there were differences in the level of social integration based on different individual, household, social, and migration characteristics. Stepwise multiple linear regression analysis was used to test the correlation between the SMI’s location and the social integration level in the overall sample. Considering self-selection bias among ERM, the propensity score method was used for robustness to minimize possible heterogeneity in personal attributes between host city and hometown SMI in ERM.

The social integration status of the experimental and control groups was compared using propensity values. The propensity value is an estimate of the probability that ERM will be insured in their host city or their hometown, given the control variables. A logistic regression model was used to estimate the sample’s propensity value for participation in the host city. Based on propensity-matched scores, we found controls of a similar nature from the control group for the experimental group. The average treatment effect (ATT) of the SMI of host city on social integration was estimated. The model was developed as follows:

1 $$P\left({X}_{i}\right)=pr\left({S}_{i}=1|{X}_{i}\right)$$

2 $$ATT=E\left({Y}_{i}|{S}_{i}=1,X=x\right)-E\left({Y}_{0}|{S}_{i}=0,X=x\right)$$

In Eq. (1), $\mathrm{P}\left({\mathrm{X}}_{\mathrm{i}}\right)$ is the propensity value. $Si$ is the independent variable, which takes the value of 1 if the residence is insured in the host city and 0 otherwise; and ${X}_{i}$ is a set of covariates. In Eq. (2), $Yi$ and ${Y}_{0}$, indicate the degree of social integration of the same ERM insured in the host city and hometown, respectively. In this study, K-neighbor matching (k = 4, caliper = 0.02) was used for analysis, and Radius caliper matching (caliper = 0.01) and Kernel caliper matching (caliper = 0.01) were used to further verify stability.

## Results

### Demographic and other characteristics

Of the 3333 samples surveyed regarding individual characteristics, age was clustered in the 60–65 age group, 60.1% were male, 65.6% had a primary school education and below, and self-assessed health accounted for 77.7%. In terms of social and family characteristics, 81.3% of employment was as employees, nearly half of the total average monthly household income was ≤ 2,999 yuan, only 28.8% had a health record, and 79.8% did not express short-stay intention in the host city, but 63.1% were willing to settle down. Regarding migration characteristics, 50.3% of the reasons for migration were for employment and 39.6% were to move in with family members; 42% of the migration occurred within the past 5 years. Regarding the migration range, 42.3% of migration was inter-provincial and 57.7% was intra-provincial. Of the ERM in the study area, 606 were insured in host city and 2,727 in their hometown.

### Social integration status

The results of the one-way analysis of variance showed that the social integration of ERM had different characteristics. Through the analysis, in addition to gender, education level, and marital status, the location of SMI, health status, average household monthly income, short-stay intention, settlement intention, term of labour contract, reasons for unemployment, employment identity, health record, migrant range, migration time and migration reason all had statistical significance. The average of the comprehensive index of social integration of ERM was -3.3 × 10^–5^, and the mean value of the comprehensive index with SMI of host city was 6.649, significantly higher than that with SMI of hometown for ERM (-1.438) (see Table 3 for details).

**Table 3:** Description of social integration of ERM with different characteristics (*n* = 3333)

**Variable name**	**Example number (person)**	**Scale (%)**	**The composite index of social integration is (**$\overline{\mathbf{X}}\pm \mathbf{S}\mathbf{D }$**)**	***F***	***P*value**
**The location of SMI**					
SMI of hometown	2727	81.8	-1.438 ± 32.795	28.311	0.000
SMI of host city	606	18.2	6.649 ± 34.383		
**Gender**					
Man	2002	60.1	0.0912 ± 33.394	0.038	0.845
Woman	1331	39.9	-0.138 ± 32.979		
**Education level**					
Primary school and below	2187	65.6	0.465 ± 33.274	1.288	0.276
Junior middle school	895	26.9	-1.487 ± 33.849		
High school and above	251	7.5	1.253 ± 30.373		
**Health status**					
Healthy	2589	77.7	-0.907 ± 33.001	8.667	0.003
Unhealthy	744	22.3	3.157 ± 33.823		
**Marital status**					
Get married, remarry and live together	2795	83.9	0.439 ± 33.099	3.030	0.082
Unmarried, divorced, or widowed	538	16.1	-2.283 ± 33.805		
**Average household monthly income (RMB)**					
≤ 2999	1675	50.3	2.898 ± 33.477	9.541	0.000
3000–5999	815	24.5	-1.604 ± 33.161		
6000–8999	455	13.7	-3.686 ± 33.517		
≥ 9000	388	11.6	-4.820 ± 30.728		
**Short-stay intention**					
Willing	2659	79.8	2.322 ± 32.830	65.440	0.000
Unwilling	674	20.2	-9.159 ± 33.210		
**Settlement intention**					
Willing	1229	36.9	7.570 ± 32.874	104.189	0.000
Unwilling	2104	63.1	-4.422 ± 32.632		
**Term of labour contract**					
No fixed term	165	5.0	-9.295 ± 35.656	14.770	0.000
Fixed term	3168	95.0	-4.479 ± 31.556		
**Reasons for unemployment**					
Family factors	457	13.7	-2.273 ± 33.556	10.057	0.000
Occupational environment factor	82	2.5	9.896 ± 34.066		
Personal physical, psychological and Other factors	2286	68.6	4.663 ± 33.410		
Non-labor force stage and other	508	15.2	1.552 ± 33.422		
**Employment identity**					
Employee	2710	81.3	-4.921 ± 32.458	13.936	0.000
Employer or operator	623	18.7	-0.768 ± 32.415		
**Health record**					
Yes	959	28.8	3.762 ± 33.458	7.030	0.000
No	1898	56.9	-3.3 × 10^–5^ ± 33.219		
Unclear	476	14.3	-2.001 ± 35.450		
**Migrant range**					
Trans-provincial	1409	42.3	-2.983 ± 33.839	9.891	0.000
Inter-city within the province	1166	35.0	2.198 ± 33.469		
The city crosses the county	758	22.7	2.164 ± 31.244		
Cross border	0	0	0		
**Migrant time (years)**					
≤ 5	1401	42.0	-4.215 ± 32.306	22.256	0.000
5–10	802	24.1	1.041 ± 34.300		
≥ 10	1130	33.9	4.487 ± 32.952		
**Migrant reason**					
Employment	1677	50.3	-1.283 ± 33.034	5.887	0.000
Family members move	1321	39.6	-0.033 ± 32.960		
Relocation and move	43	1.3	19.761 ± 36.876		
To grow old	236	7.1	4.324 ± 32.505		
Other	56	1.7	5.791 ± 39.063		

*SD* Standard deviations

### Relationship between social integration, place of enrollment, and other variables

The stepwise multiple linear regression was based on the set of independent variables in Table 2, excluding variables that were not statistically different in the one-way analysis of variance. The excluded variables were gender, education level, employment identity and marital status. The results of the multiple linear regression showed that the location of SMI was significantly and positively associated with the level of social integration (coefficient = 3.916, *p* < 0.01). Higher levels of social integration were associated with location of SMI. Higher levels of social integration were associated with host city SMI than hometown SMI. Among individual characteristics, health status (coefficient = 0.016, *p* > 0.10) was not statistically significant. Among household characteristics, average household monthly income was negatively associated with social integration (coefficient = -1.979, *p* < 0.01). Among the ERM, settlement intention (coefficient = -9.846, *p* < 0.01) and short-stay intention (coefficient = -6.334, *p* < 0.01) had a significant negative correlation with the level of social integration. No intention to settle and no short-stay intention were associated with a higher level of social integration. The level of social integration of people with no settlement intention and no short-stay intention was higher than that of people with settlement intention and short-stay intention. Among the social characteristics, term of labour contract was not statistically significant (coefficient = 0.022, *p* > 0.10). Employment identity was not statistically significant (coefficient = 0.03, *p* > 0.10). Reasons for unemployment was not statistically significant (coefficient = 0.003, *p* > 0.10) (see Table 4 for details). Health record was negatively associated with social integration (coefficient = -2.241, *p* < 0.05). Among migrant characteristics, migrant range (coefficient = 2.659, *p* < 0.01), migrant time (coefficient = 2.908, *p* < 0.01) and migration reason (coefficient = 1.838, *p* < 0.01) are all positively associated with social integration.

**Table 4:** Stepwise multiple linear regression analysis of the relationship between location of SMI and social integration among ERM (*n* = 3333)

**Model 8**	**Unstandardized coefficients**	**Standard error**	**Standardized coefficients**	**t**	***P* value**	**95% confidence interval**
**(Constant)**	17.290	3.973		4.352	0.000	(9.501, 25.080)
**Location of SMI**	3.916	1.495	0.045	2.620	0.009	(0.985, 6.848)
**Settlement intention**	-9.846	1.222	-0.143	-8.054	0.000	(-12.242, -7.448)
**Short-stay intention**	-6.334	1.465	-0.077	-4.323	0.000	(-9.207, -3.462)
**Average household monthly income**	-1.979	0.549	-0.062	-3.602	0.000	(-3.056, -.902)
**Health record**	-2.241	0.878	-0.043	-2.553	0.011	(-3.961, -.520)
**Migrant time**	2.908	0.675	0.076	4.311	0.000	(1.586, 4.231)
**Migrant range**	2.659	0.734	0.063	3.624	0.000	(1.220, 4.097)
**Migrant reason**	1.838	0.615	0.051	2.987	0.003	(0.631, 3.044)

### Robustness tests

Matching was performed using k-nearest caliper matching (*n* = 4, caliper = 0.02), and the differences in variables before and after matching are shown in Table 5. For k-nearest caliper matching (*n* = 4, caliper = 0.02), the full sample ATT value was 6.47 (t = 5.32, standard error = 1.48, *p* < 0.05), indicating that the SMI of host city increased the probability of the social integration index by 647%. To further ensure robustness, the treatment effect was tested using the radius caliper and kernel matching methods. Using the radius caliper method, the ATT value for the full sample was 6.52 (t = 5.32, SE = 1.49) when caliper = 0.01. This indicates a 652% increase in the social integration index of ERM. In the kernel matching method, when caliper = 0.01, the ATT value for the whole sample is 6.47 (t = 5.32, SE = 1.49). The social integration level is higher with SMI of host cities than that of hometowns. In conclusion, SMI of host cities can significantly contribute to the social integration level of ERM. The results of this paper are robust. The average treatment effects of place of participation on social integration under multiple matching methods are detailed in Table 6.

**Table 5:** Variation of variable before and after propensity score matching (*n* = 4, caliper = 0.02)

**Variable**	**Mean**		***SD***	**Deviation reduction**	**t**	***P* value**
	**SMI of host city**	**SMI of hometown**				
**Migrant range**						
Match before	1.71	1.83	-15.6	90.70	-3.43	0.001
After matching	1.71	1.73	-2.80		-0.26	0.796
**Average household monthly income**						
Match before	1.66	1.91	-25.5	75.00	-5.47	0.000
After matching	1.66	1.60	6.40		1.19	0.233
**Short-stay intention**						
Match before	1.11	1.22	-29.20	86.10	-6.02	0.000
After matching	1.11	1.12	-4.10		-0.80	0.425
**Settlement intention**						
Match before	1.51	1.66	-29.60	83.60	-6.70	0.000
After matching	1.52	1.54	-4.90		-0.82	0.411
**Health record**						
Match before	1.74	1.87	-18.90	78.00	-4.44	0.000
After matching	1.75	1.71	4.20		0.70	0.485
**Migrant time**						
Match before	2.26	1.84	48.70	94.80	10.86	0.000
After matching	2.26	2.28	-2.50		-0.44	0.661
**Migrant reason**						
Match before	1.79	1.68	11.50	38.10	2.70	0.007
After matching	1.78	1.71	7.10		1.24	0.216

*SD* Standard deviations

**Table 6:** The processing effect of propensity score matching

**Methods**	***ATT***	***SD***	***z***	***P* value**
K-neighbor matching(k = 4, caliper = 0.02)	6.47	2.017	1.960	0.049
Radius caliper l matching(caliper = 0.01)	6.52	1.749	2.010	0.003
Kernel caliper matching (caliper = 0.01)	6.47	1.635	2.350	0.019

*SD* Standard deviations

## Discussion

To the best of our knowledge, this is the first study to examine the relationship between social integration and location of SMI in a sample of ERM. The main findings of the study are as follows. First, the level of social integration is significantly related to the location of SMI. However, social integration is not related to individual characteristics, but is mixed with family, social, and migration characteristics. Social integration is negatively correlated with average household monthly income, short-stay intention, and settlement intention. Second, higher levels of social integration are associated with host city SMI, while ERM with SMI of hometown have lower integration levels. Promoting direct coverage of host city SMI for ERM would promote the social integration and improve their well-being.

There is a large gap between the population size of SMI host cities and hometowns. The results of this study show that 606 people, or 18.2%, chose to enroll in host cities, while 2727 people, or 81.8%, chose to enroll in hometowns. The possible reason is that migrants are not eligible to register in host cities. First, without household registration, it may not be possible to enroll in insurance. Qian’s study concluded that the lack of local household registration may lead to difficulties for migrants to enroll in host city SMI [36]. The lack of household registration prevents ERM from receiving the same benefits as natives. Second, they do not meet the requirements for a residence permit to enroll in SMI. In addition, the reason for choosing hometown SMI to enroll may be that they are familiar with the SMI policies there. The simplicity of the enrollment process in hometowns make the ERM more willing to choose it.

Our study found that social participation is a key component of social integration. The analysis of the data in this paper shows that the social integration of ERM consists mainly of social participation, governance participation, psychological identification, and custom participation. The results of the study showed that the contribution of social participation was 45.42%, while the contributions of custom participation, psychological identification and governance participation were 20.61%, 20.61% and 19.61%, respectively. This is similar to the results of previous studies on social integration. Immigration Policy Lab presented the Immigration Policy Lab Integration Index in 2018, which evaluates six dimensions that include social participation, governance participation, psychological identity [28]. Moreover, customs participation is an important area to study integration [1, 37, 38]. For ERM, identifying with the culture of host cities is a challenge. This is because ERM usually have deep-rooted beliefs about their home culture. A large amount of contemporary research on cultural integration confirms that cultural acceptance promotes social integration more than cultural naturalization and cultural identity [39]. Therefore, we suggest that local communities can organize activities that respect and accept their home cultures to promote the social integration of ERM.

Our study found that the social integration level of ERM varies according to family characteristics, social characteristics, and migration characteristics. The relationship between household characteristics and social integration is as follows. First, the higher average household monthly income, the lower the social integration level. In an article on the socioeconomic status and happiness of migrants, it is said that among population with higher levels of social income, the happiness of migrants without household registration is lower than that of people with household registration [40]. The possible reason is that the perceived happiness of the high-income group is more influenced by institutional identity. Second, the integration level is higher for the groups with low short-stay intention and settlement intention. This is contrary to the findings of previous all-age studies [41]. As ERM are all above 60 years old, they tend to have unstable jobs, and have more difficulties settling in host cities. Therefore, we suggest that policies and social services for ERM who have lived longer should be strengthened to promote higher social integration. Again, this study does not show that marital status is related to social integration. The reason may be that the social integration of ERM is less influenced by the marital relationship. The marital relationship is no longer the main social relationship. The relationship between social characteristics and social integration is as follows. First, in the ERM, the integration level with health records is lower than that without health records. That is, the ERM without health records have a higher social integration level. This contradicts the results of previous studies [19, 42, 43]. The possible reason for this is that the ERM without health records has a better health status. On the other hand, the creation of health records is often done during medical visits. Poor health status may be associated with low social integration. Second, term of labour contract, reasons for unemployment, employment identity and the employment status were not related to the social integration level. This may be explained by the fact that the ERM in our study were all over 60 years old and often had unstable jobs. Therefore, social integration was not significantly influenced by the above three characteristics. The relationship between migration characteristics and social integration is the following. First, the greater migration range, the higher the social integration level. The longer the duration of migration, the higher the social integration level. The possible reason for this is that the greater the range of migration and the longer the duration of migration, the less the emotional attachment to the hometown and thus better integration into the host cities. Second, ERM moved for family do not have as high an integration level as those who move for employment. Previous studies have argued that ERM’s babysitting does not increase integration level. The lack of effective companionship does not promote the social integration of ERM who are involved in babysitting and household chores [31, 44]. However, it has also been argued that family-based migration promotes community integration [45]. Considering that this study does not exclude the possibility of following family migration for health reasons. Such elderly people may face health problems that prevent them from self-care. In turn, the inability to take care of themselves will hinder social integration. Therefore, it is hoped that the relationship between different situations of family migration and social integration can be further explored in future studies.. For groups that are migrant for employment reasons, work provides ERM with access to multiple forms of social interaction.

Our study shows that a high level of social integration is associated with host city SMI. The social integration level of ERM with hometown SMI is lower compared to those with host city SMI. Specifically, host city SMI is significantly and positively associated with social integration. After balancing the control variables, the social integration level is higher for those with host city SMI than those with hometown SMI. It indicates that the location of SMI will have a non-negligible impact on the integration level for ERM. Possible reasons for this are as follows. First, there is ample evidence that migrants with host city SMI are more familiar with the host city health care system and health policies [46, 47]. They are more likely to choose to receive health care there. This is to some extent consistent with our findings. Perhaps this is because host city SMI improves the economic accessibility of health care by providing subsidies. Second, host city SMI facilitates social interaction and thus enhances integration. Specifically, in the process of enrolling and receiving health care, the interactions between participants and health care providers increased. Okamoto’s study concluded that increased social interactions helped to reduce social isolation [48]. Relatively speaking, hometown SMI was associated with lower social integration. Enrolling hometown SMI means that one either has to return to the hometown for health care and subsidies, or be reimbursed off-site in host cities. First, there is evidence that migrants with hometown SMI are more likely to return to their hometown for health care. This is consistent with previous research on internal migration [49]. Now, the SMI systems across China are not interoperable. Specifically, if the location of SMI and health care place do not coincide, participants will face a cumbersome reimbursement process and potentially less reimbursement. And the ERM without host city SMI often choose their hometown for subsidization. Maybe it is because they are more familiar with the healthcare in hometown. The inability of host cities to meet the demand for healthcare is not conducive to the social integration. Second, there is a difference between off-site reimbursement and reimbursement at the place of enrollment [50]. Off-site reimbursement implies limited reimbursement coverage, uncertain reimbursement rates, and filing procedures. However, policymakers are constantly pushing for regional integration of reimbursement systems in order to eliminate the differences in off-site reimbursement within the region. But now, the policies of regional integration are only realized in individual regions. Limited reimbursement coverage, uncertain reimbursement rates, and filing procedures all set barriers to social integration. Therefore, our study suggests promoting the direct enrollment of ERM in host cities. Song’s study on the household registration system suggests that policy adjustments would be more beneficial than other measures to promote the social integration of migrants [42]. This is consistent with our view. We advocate that policymakers focus on the access of host city SMI for ERM. Eliminating the host city SMI threshold promotes social integration.

### Limitations

First, the indicators of social integration in the questionnaire may have certain limitations. Although the questionnaire is comprehensive in what it covers, it may still lack content related to social integration, such as the lack of the presence of dialectal communication. One example is whether the dialect of the migrant population is significantly different from that of the host city to the extent that it causes communication barriers. Then, SMI policies are not identical across different host cities in China. This study has not yet delved into the specific impacts of policy differences. It is hoped that subsequent studies can explore further. Further, there is less research on the different types of insurance policies available in the host cities. As health insurance policies are continuously updated, its mechanisms need to be further studied in order to provide timely policy recommendations. Finally, this study was a cross-sectional design, so causality cannot be determined.

## Conclusions

We identified that ERM with host city SMI have a higher social integration level compared to those with hometown SMI. That is, host city SMI has a positive effect on the social integration of ERM. Policymakers could focus on the access of host city SMI for ERM. Eliminating the host city SMI threshold promotes social integration. In addition, we find that the amount of people with host city SMI is much lower than that with hometown SMI. Our results indicate that social participation contributes to integration more than other factors.

## Data Availability

The datasets generated and/or analysed during the current study are available in the (Migrant Population Service Center, National Health Commission P.R. China repository, https://www.chinaldrk.org.cn/wjw/#/home).

## Abbreviations

ATT: Average treatment effect on the treated
ERM: Elderly rural migrants
SD: Standard deviations
SMI: Social medical insurance
